# Cancrum Oris

**Published:** 1872-05

**Authors:** C. S. Kittredge


### ARTICLE VIII.

### *Paper on Cancrum Oris.*

BY C. S. KITTREDGE, M.D.

During the years 1863–'64, it was my privilege to be the assistant of the late and much lamented Henry Whittelsey, M. D., then the resident physician at the Nursery Hospital, on Randall's Island, New York city.

It was our fortune to have during the year, a large number of cases of cancrum oris, and our conclusions with reference to this peculiarly fatal disease may be of interest to the profession.

This formidable affection—which is a malignant form of Ulcerative Stomatitis—is a disease of debility, induced in children under five years of age, predisposed to tuberculosis by a want of cleanliness, proper food, and care; and appears chiefly in those who are received into our hospitals from the alm-houses, jails and poorly ventillated lodgings—those dark, damp, filthy apartments, where congregate the vice and destitution of our large cities.

It appears generally as the sequel to rubeola, scarlatina, or some other debilitating disease. It occurs at all seasons of the year, but is most prevalent during the summer months.

That it is a contagious disease, though in a limited degree, cannot be doubted, yet I believe that only those who are badly nourished, and of a tuberculous or scrofulous diathesis, are liable to be affected.

*Mode of access.* The system being in a morbid state, a simple shallow ulcer makes its appearance on the inside of the cheek, or between the two incisor teeth of the upper jaw, at the junction of the frænum labii superioris with the gums, causing the lips to swell rapidly and enormously, the teeth to loosen and drop out, and a general destruction of the parts; or, it may commence in the mucous membrane of the throat, or roof of the mouth, destroying everything in its course.

A description of this disease as it appears and progresses when commencing in the cheek—its most common form—will suffice to show all its characteristic features. The first thing noticeable, is a slight circumscribed swelling, hard, painless, red, tense and shining, resembling somewhat the sting of a bee. A very small whitish ulcer will now be found on the inside of the cheek, corresponding in position with the centre of the external induration. The breath is slightly fœtid and an increase in the secretion of saliva is apparent. The ulcer very soon becomes ash-colored, and rapidly spreads, assuming a phagedenic character, discharging an offensive dirty-colored fluid. In two or three days, a black depressed spot appears on the outside of the cheek, corresponding in size and position with the ulcer within. In the course of another day, a small hole is seen in the centre of this gangrenous spot, through which ooze, drop by drop, the offensive acid discharge. The face now assumes a peculiar ashy-yellow color, swells greatly, frequently to such an extent as to close both eyes; mortification goes on rapidly, often involving the whole cheek, and exposing the cavity of the mouth. The bones of the face may become affected with necrosis, the teeth fall out, and the little patient becomes a most frightful spectacle to behold, while he horrible stench that arises is well nigh unbearable

The constitutional symptoms are extreme prostration, with rapid, feeble pulse, increasing as the disease advances. The mind is usually clear to the last, yet the little patient lies in that listless and indifferent condition, so common with typhoid patients, and dies from sheer exhaustion.

The duration of this disease is from six to sixteen days, running a most rapid course when commencing in the cheek or throat, but somewhat slower in its progress when commencing in the gums.

*Diagnosis.* This can be mistaken for no other disease. Its first stage is that of ulceration stomatitis; but it differs from the simple form of this disease in the marked and constantly increasing debility of the patient. It also speedily assumes its diagnostic phagedenic character, becoming gangrenous usually in two or three days from its inception.

*Treatment.* This disease demands prompt and energetic treatment. As soon as an ulcer shows itself, I commence with the chlorate of potassa, in from five to ten grain doses every four to six hours, and continue the administration of this medicine—which is justly considered almost a specific in the simple form of stomatitis—during the whole progress of the disease. We must not, however, rely wholly upon this remedy. Our chief hope lies in preventing the ulcers from assuming a phagedenic character, and to meet this end, the part should be thoroughly cauterized as soon as any signs of sloughing appear. The child should be imme diately transferred to a room separate from all other children, and every care taken that the disease be not communicated to others, and all cases of stomatitis then in the hospital should be at once cauterized, even before assuming the characteristic malignant features. Of all the caustics, I prefer the solid nitrate of silver, for most cases; yet it will often be necessary to use the acidnitrate of mercury, strong hydrochloric acid, bromine, or other powerful caustics; and even then we too often fail in our attempts to prevent the rapid spread of the disease. The mouth must be frequently washed with a weak solution of liquor sodæ chlorinatæ, ℥j to water ℥xij, and after mortification has commenced, a pledget of soft linen wet in this solution should be constantly kept between the sore and the adjacent tissue. Tonics and stimulants should be freely given on account of the great prostration, and iron with bark or quinine in as heavy doses as the child will bear, and strong beef tea in place of solid food, which latter the child will almost invariably refuse to take, are the best.

*Prognosis.* Cancrum oris must be regarded as a peculiarly fatal form of stomatitis; in fact, every case that came under our observation, fourteen in number, when the disease was not checked before becoming gangrenous, died. Out out of sixty-seven cases of stomatitis treated during the year, there were nineteen deaths, or twenty-eight per cent. This includes not only every case that was regarded as cancrum oris, but five others which, having the same characteristic features, proved fatal before the disease reached the gangrenous state.

The following report of cases, taken from my note book, may not be without interest:

*Case* 1. C. S., aged two and a half years, was admitted to the hospital, April 28th, with rubeola, having pneumonia as a complication. He recovered so far as to be up and playing in the convalescent ward, yet he did not gain strength.

June 10th. A small ulcer made its appearance at the junction of the frænum labii superioris with the gums, just between the two incisor teeth. I touched this ulcer with the solid nitrate of silver, and gave internally the chlorate of potassa in five grain doses every four hours, in addition to the tonic of quinine and iron that he had been taking for three weeks.

June 11th. The ulceration spreading. The two incisor teeth being very loose, I took them out of the way and again applied the nitrate of silver thoroughly to the ulcer.

June 12th. Upper lip much swelled; ulceration worse; applied bromine to the part. Wash the mouth frequently during the day and night with the following: ℞ Potass. Chlorat, ʒ iv; Tr. Myrrhæ, ℥ j; Tr. Ferri Chl., ʒ iij; Syr. Aurant, ℥ iv; Aquæ, ℥ ij*ss*; *M.*

June 15th. Both cheeks swollen; upper lip protrudes; has lost another tooth. Bromine has been applied daily, and a cloth wet with liquor sodæ chlorinat. diluted with ten parts of water, kept constantly under the lip.

June 16th. The ulceration has eaten through the lip at the junction of the right ala of the nose, making a black gangrenous spot about the size of a dime. The lip is fully an inch thick.

June 18th. The gangrene extends about two inches in length, by an inch in width, including the end of the nose; the face is of a peculiar ashy-yellow color; both eyes are closed by the swelling; he is extremely weak.

June 21st. Died last evening. The mortification extends in a circle over three inches in diameter, destroying the whole nose, and including most of the upper lip. An autopsy revealed abundance of miliary tubercles throughout both lungs.

*Case* 2. J. T., aged two years, was admitted to the hospital, May 22d, with a light attack of rubeola. He gained no strength, and did not wish to leave his bed. During the first week in June, he passed by the mouth and nose, a number of worms—ascarides lumbricoides. He also had diarrhœa.

June 11th. His mouth is just beginning to be sore; a small ulcerated point is visible at the edge of the frænum labii superioris, at its junction with the gums; the gums are slightly inflamed and swollen. Touched the ulcer with nitrate of silver, and frequently during the day washed the mouth thoroughly with the following: ℞ Potass. Chlorat, ʒ iv; Tr. Myrrhæ, ℥ j; Tr. Ferri Chl., ʒ iij; Syr. Aurantii, ℥ iv; Aquæ, ℥ ij*ss*; *M.* Gave internally chlorate of potassa in five grain doses, every four hours, in addition to the tonic of quinine and iron which he has been taking for some days.

June 12th. The ulceration is spreading along the upper jaw, and the inflammation has extended to the gums of the lower.

June 14th. Mouth worse. Changed the wash to: ℞ Cupri Sulph., ʒ ij; Cinchonæ Pulv., ℥ *ss;* Aquæ, ℥ iv; *M.*, and apply with a brush.

June 15th. This morning I noticed for the first time, on the left cheek, a small red spot, about the size of a pin's head, in the centre of a large red spot about the size of a quarter of a dollar. The cheek is much swollen, and on the inside, corresponding with the smaller spot, is an ulcer about the size of a dime, ash-colored, gangrenous, and emitting its peculiar offensive odor. Ordered the nurse to wash the mouth thoroughly with liquor sodæ chlor., diluted with four parts of water, and a cloth wet with this solution to be kept constantly between the jaw and the cheek.

June 16th. The small spot on the outside of the cheek has to-day the appearance of an ulcer. Cheek much swollen, hard and livid.

June 22d. The swelling has gradually diminished. A black spot has this morning made its appearance on the left side of the nose, an inch and a half in length by half an inch in width, extending to the middle of the lip. It is of a bluish-black color, and slightly sunken from the rest of the face. Have poulticed the face to-day.

June 26th. Died this morning. The gangrenous portion has gradually increased in size, covering most of the left cheek and lip. No autopsy allowed.—*Western Lancet.*

---

